# Supplementary material for: Spontaneous variability in gamma dynamics described by a damped harmonic oscillator driven by noise
Source: Nat Commun. 2022 Apr 19;13:2019. doi: 10.1038/s41467-022-29674-x (PMC9018758; doi:10.1038/s41467-022-29674-x)
Supplement: Supplementary file 1 — Supplementary Information [file 41467_2022_29674_MOESM1_ESM.pdf]

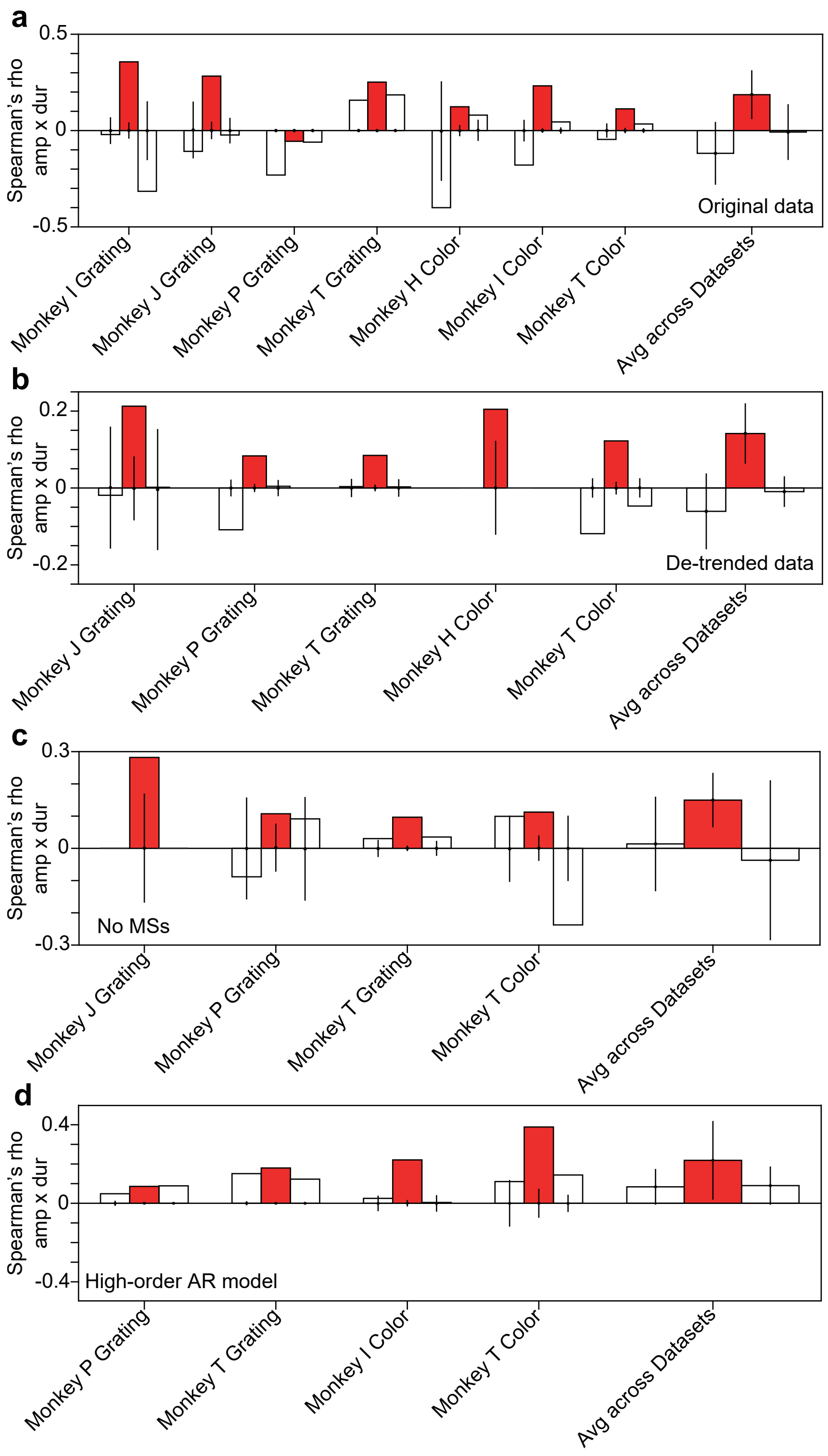

Supplementary Figure 1

Supplementary Figure | 1. Gamma-full-cycle amplitudes and durations are positively correlated. (**a**) Same as **Fig. 4a**, but using full gamma cycles (numbers of trials = 278, 5740, 672, 1075, 672, 320, 142 for respective datasets; averages across datasets: red bar: p=0.011, 2-sided t-test across datasets; p<0.05, two-sided randomization test across datasets; white bars p=0.13 and p=0.9, respectively for preceding and succeeding cycles, 2-sided t-test across datasets; p>0.05, two-sided randomization test across datasets). n=6 biologically independent samples. (**b**) Same as **Fig. 4c**, but using full gamma cycles (numbers of trials = 5740, 672, 1075, 672, 142 for respective datasets; averages across datasets: red bar: p=0.008, 2-sided t-test across datasets; white bars p=0.15 and p=0.51, respectively for preceding and succeeding cycles, 2-sided t-test across datasets). n=5 biologically independent samples. (**c**) Same as **Supplementary Figure 2c**, but using full gamma cycles (numbers of trials = 672, 1075, 320, 142 for respective datasets; averages across datasets: red bar: p=0.046, t-test across datasets; white bars p=0.11 and p=0.13, respectively for preceding and succeeding cycles, 2-sided t-test across datasets). n=4 biologically independent samples. (**d**) Same as **Supplementary Figure 3e**, but using full gamma cycles (numbers of trials = 5740, 672, 1075, 142 for respective datasets; averages across datasets: red bar: p=0.041, 2-sided t-test across datasets; white bars p=0.9 and p=0.7, respectively for preceding and succeeding cycles, 2-sided t-test across datasets). n=4 biologically independent samples. Source data are provided in the Source Data file.

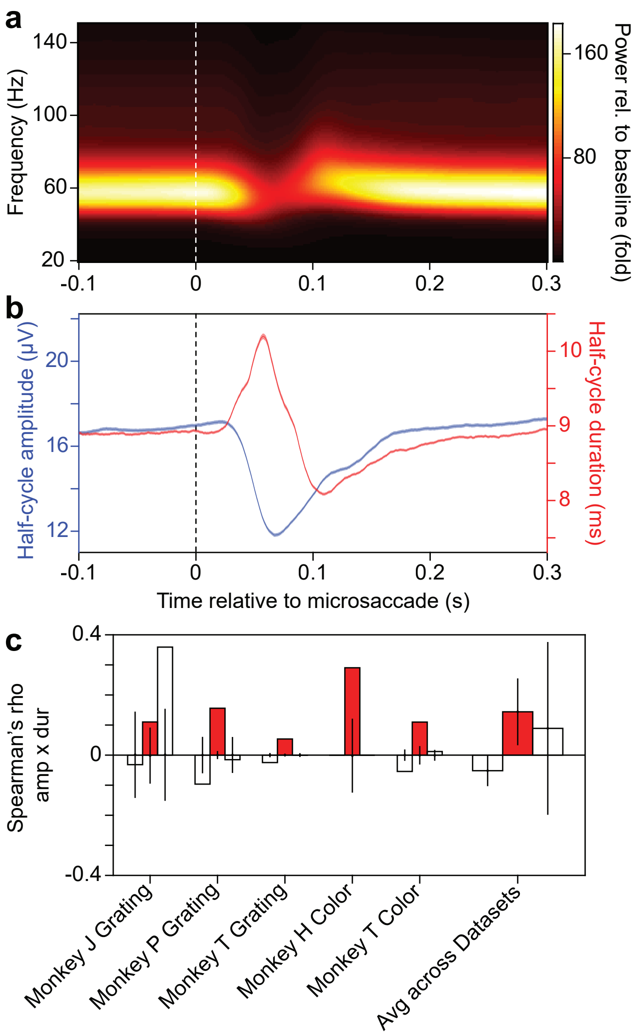

Supplementary Figure 2

Supplementary Figure | 2. The effect of microsaccades on the correlation between gamma-half-cycle amplitudes and durations. (**a**) Time-frequency power averaged over all selected V1 recording sites in monkey T during the presentation of a full-screen drifting grating, normalized by the pre-stimulus baseline. X-axis shows time relative to detected microsaccades (MSs). (**b**) Time-course of the gamma-half-cycle amplitude (blue) and duration (red) of the data depicted in **a**. Error regions show ±2 SEM based on a bootstrap over MSs. (**c**) Same as **Fig. 4c**, but after the removal of 250 ms epochs following the occurrence of MSs for all available datasets (numbers of trials = 5740, 672, 1075, 672, 142 for respective datasets; averages across datasets: red bar: $p=0.02$, 2-sided t-test across datasets; white bars $p=0.07$ and $p=0.97$, respectively for preceding and succeeding cycles, 2-sided t-test across datasets). $n=5$ biologically independent samples. Source data are provided in the Source Data file.

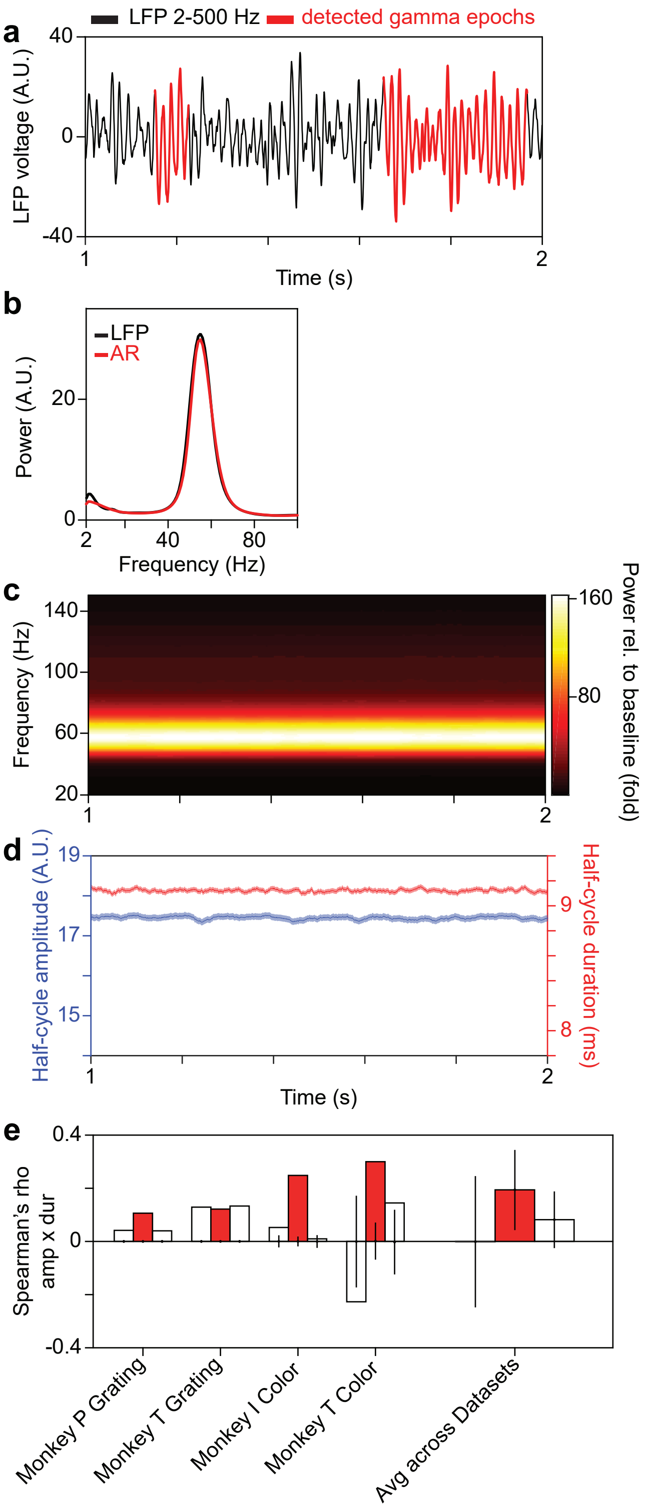

Supplementary Figure 3

Supplementary Figure | 3. Correlation of gamma-half-cycle amplitudes and durations in an AR model of the visual stimulation period. Panels (**a**-**d**) are based on signals generated by an autoregressive (AR) model of the data used in **Fig. 1a**-**d**, for the visual-stimulation period, averaged over all selected V1 sites. We refer to the synthetic LFP signal generated by the AR model as AR-based LFP. (**a**) Representative AR-based LFP. Regions presented in red correspond to gamma epochs passing the criterion for stationarity. (**b**) Average raw power of the measured (black) and the AR-based LFP (red). (**c**) Time-frequency power of AR-based LFP. Note the expected absence of temporal trends. (**d**) Time-course of gamma-half-cycle amplitude (blue) and duration (red) of AR-based LFP. Error regions show ±2 SEM based on a bootstrap procedure. (**e**) Same as **Fig. 4a**, but for the AR-based LFP (numbers of trials = 672, 1075, 320, 142 for respective datasets; averages across datasets: red bar: $p=0.03$, 2-sided t-test across datasets; white bars $p=0.98$ and $p=0.2$, respectively for preceding and succeeding cycles, 2-sided t-test across datasets). $n=4$ biologically independent samples. Source data are provided in the Source Data file.

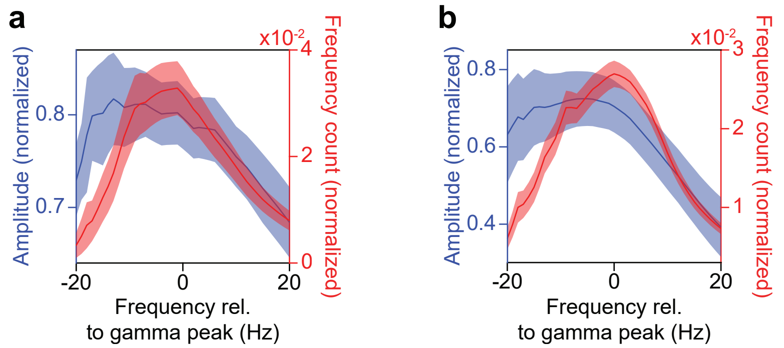

Supplementary Figure 4

Supplementary Figure | 4. Cycle-based spectra of amplitudes and rates of incidence. (**a**) Same as **Fig. 5a**, and (**b**) same as **Fig. 5b**, but after aligning to the gamma peak in the power-change spectrum. Source data are provided in the Source Data file.

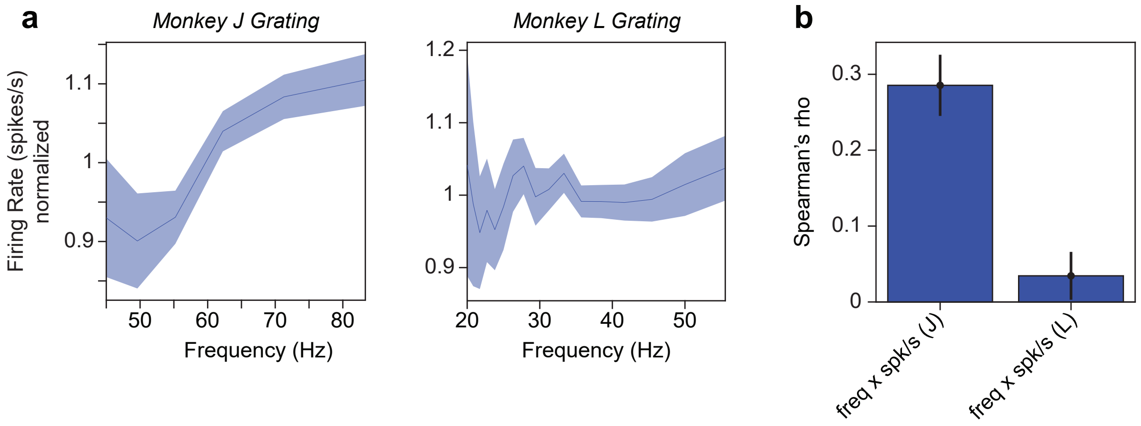

Supplementary Figure 5

Supplementary Figure | 5. The relationship between spiking and gamma-cycle trough-to-peak duration. (**a**) Same as **Fig. 6b**, but for the firing rate for the trough-to-peak period and the duration of the subsequent peak-to-trough half-cycle. (**b**) Same as **Fig. 6d** (second pair of bars from the left), but for the firing rate for the trough-to-peak period and the duration of the subsequent peak-to-trough half-cycle. a-b: n=156 and 127 independent MU channels examined over 35 and 40 independent sessions in monkey J and L, respectively. Source data are provided in the Source Data file.

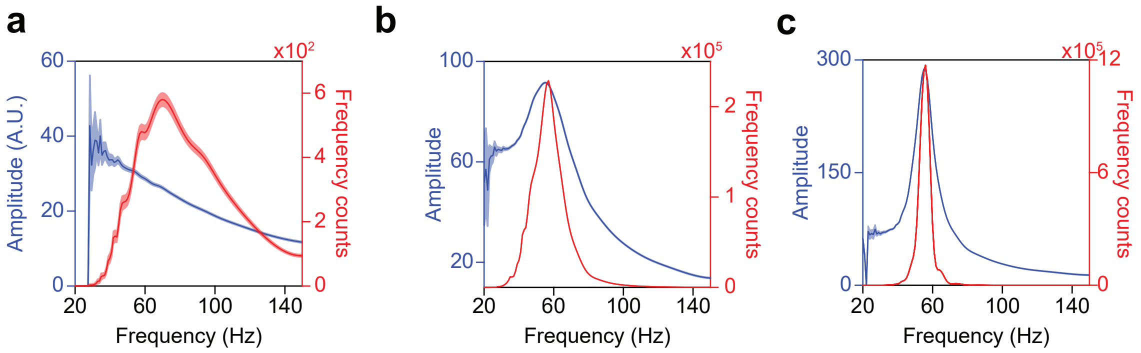

Supplementary Figure 6

Supplementary Figure | 6. Cycle-based spectra of amplitudes and rates of incidence for damped harmonic oscillators driven by noise. (**a**-**c**) Same as **Fig. 5a**, but for synthetic signals generated from AR(2) processes with respective eigenvalue magnitudes of 0.9 (**a**), 0.9871 (**b**; same as median of **Fig. 8c**), and 0.999 (**c**).

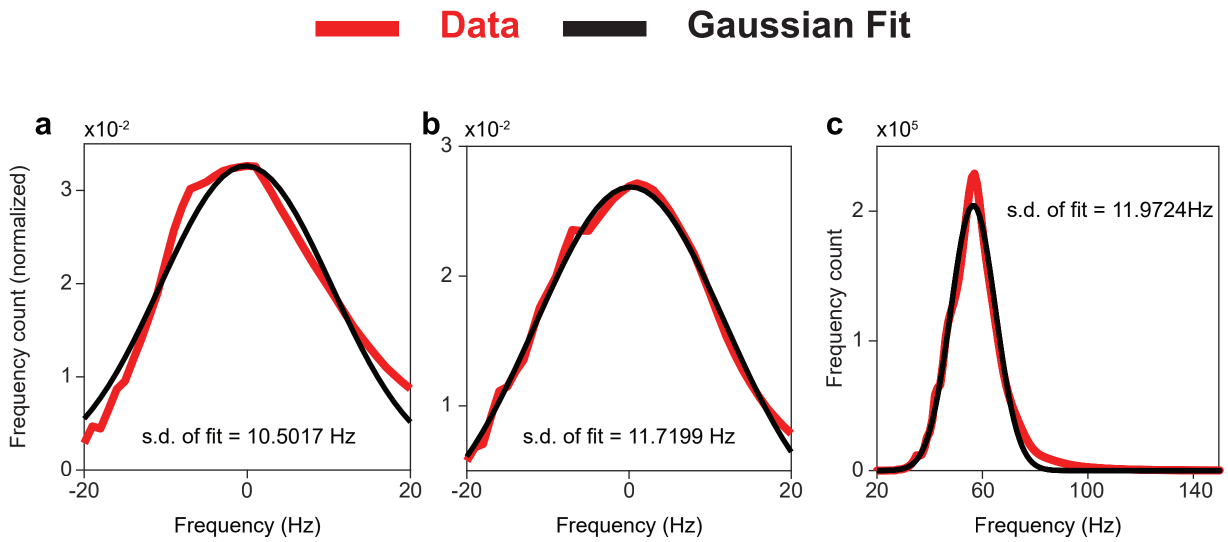

Supplementary Figure 7

Supplementary Figure | 7. Variability in cycle-based frequency rates of incidence. (**a**) Gaussian fit on the count of detected gamma half-cycles as a function of the inverse of their duration (**Fig. 5a** red). (**b**) Same as **a**, but for **Fig. 5b**. (**c**) Same as **a**, **b**, but for **Fig. 6b**. Uses the Source Data for Figure 5.

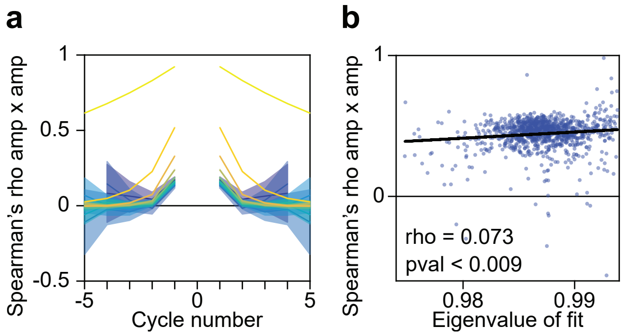

Supplementary Figure 8

Supplementary Figure | 8. A damped harmonic oscillator driven by noise reproduces LFP gamma-cycle amplitude autocorrelations estimated for full-cycles. (**a**,**b**) Same as, respectively, **Fig. 9c** and **Fig. 9e**, but for full gamma cycles. Source data are provided in the Source Data file.

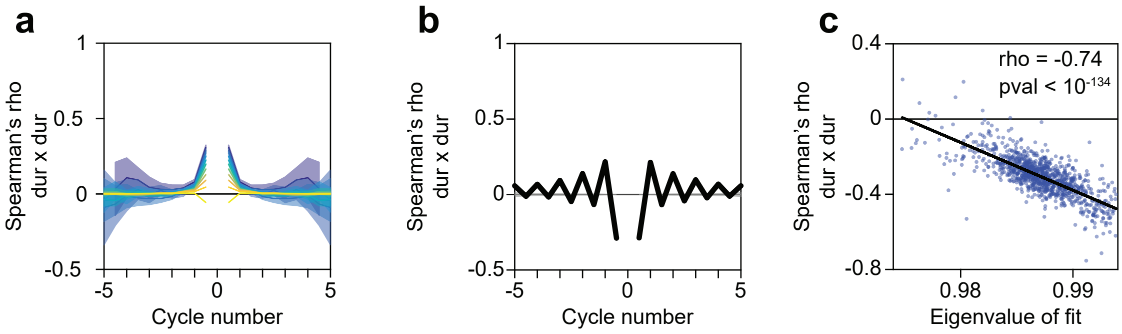

Supplementary Figure 9

Supplementary Figure | 9. A damped harmonic oscillator driven by noise reproduces LFP gamma-cycle duration autocorrelations estimated for half-cycles. (**a-c**) Same as **Fig. 9f-h**, but for gamma half-cycles. Source data are provided in the Source Data file.

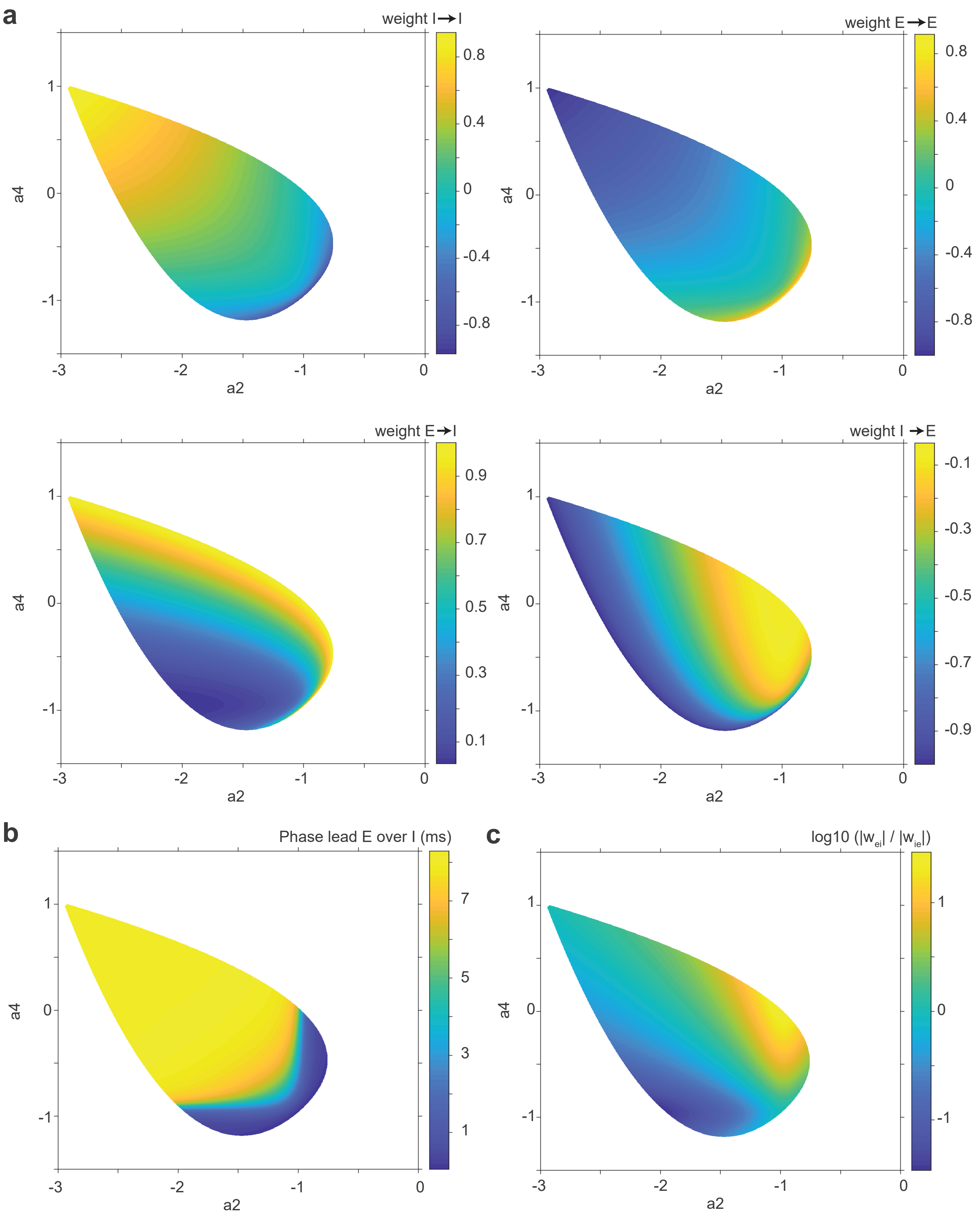

Supplementary Figure 10

Supplementary Figure | 10. Transformation of AR(2) in a linear E-I circuit forced by white noise. (**a**) Weight matrices for w_{ee} – 1, w_{ii} – 1, w_{ei}, w_{ie} as a function of the transformation coefficients a_2 and a_4 in the matrix A (see Supplementary Appendix) that maps (x_t, x_{t-1}) onto (E_t, I_t). Shown are the regions in space where all the weights have a magnitude smaller than 1. (**b,c**) Phase lead of E over I in ms at 60Hz (**b**) and the E-I balance, measured as $\log_{10}(|w_{ei}|/|w_{ie}|)$ (**c**).

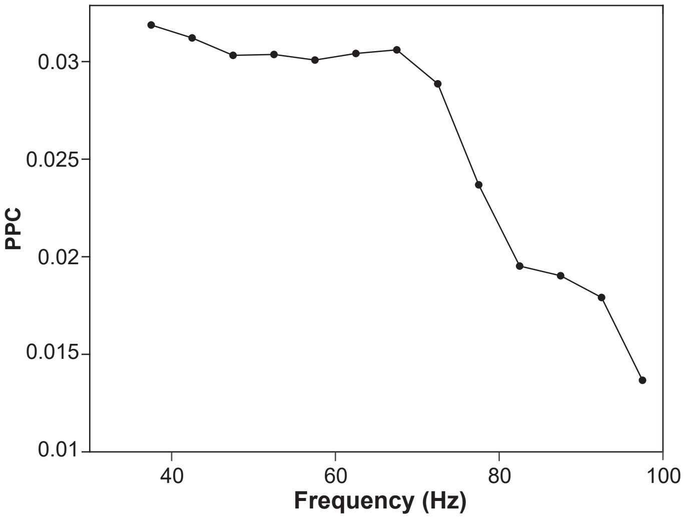

Supplementary Figure 11

Supplementary Figure | 11. Relationship between spike synchrony and gamma-cycle duration in an AR(2) model. We analyzed neurons that fired spikes according to inhomogeneous Poisson processes with a conditional intensity function that was given by the E-variable in the AR(2) model. This E variable was obtained by the equation E[t] = x[t] – 1.1 x[t-1] (see Supplementary Appendix). The E variable was transformed into a conditional intensity function by first scaling the signal between -1 and 1, yielding E_{norm}, and then defining $r(t) = r_0 + r_0 E_{norm}$. Here r_0 is the average firing rate. The resulting firing rate thus ranged between $[0,2*r_0]$. We then generated spikes for each time point according to an independent Poisson distribution, and computed the spike-field PPC between the spikes and the E variable. In total we included 10^5 neurons with an average firing rate of r_0 = 1Hz for a trace of 10^3 s long. The AR(2) model had an eigenvalue of 0.99, close to the eigenvalue magnitude in the LFP data, and a center frequency of 60 Hz. The figure shows the average phase-locking between spikes and the AR(2) E-variable, as a function of frequency. The correlation between PPC and frequency was significantly negative (rho = -0.91, p=0).

Supplementary Note 1

"Spontaneous variability in gamma dynamics described by a linear harmonic oscillator driven by noise"

Spyropoulos, Saponati et al.

March 14, 2022

We formally show the relations between AR(2) models and linear E-I circuits and we discuss how to constrain the affine transformation A to account for published literature on the E and I populations in area V1. The code to reproduce the figures in this supplementary document is available on GitHub (@matteosaponati).

Contents

1 AR(2) model **1**

2 Linear E-I circuit **2**

3 AR(2) model to E-I circuit **3**

3.1 General form of the affine weights . . . 3

3.2 Opposite sign of the coupling weights . . . 4

3.3 Affine transformation of the phase space . . . 5

3.4 Choice of the affine transformation . . . 7

1 AR(2) model

We briefly revisit the basic definition of an AR(2) model. An AR(2) is defined as a second order linear difference equation of the form

$$x_t = \beta_1 x_{t-1} + \beta_2 x_{t-2} + \epsilon_t \tag{1}$$

or, in the lag operator formalism

$$(1 - \beta_1 L - \beta_2 L^2) x_t = \epsilon_t \,,$$

where L is the usual lag-operator $L\, x_t \triangleq x_{t-1}$. The dynamics of this second-order system are completely described by the roots of the polynomial in the lag operator on the l.h.s. of the latter equation or, equivalently, by the roots of the characteristic polynomial

$$\lambda^2 - \beta_1 \lambda - \beta_2 = 0 \tag{2}$$

which are the eigenvalues of the matrix B defined by

$$\begin{pmatrix} x_t \\ x_{t-1} \end{pmatrix} = \underbrace{\begin{pmatrix} \beta_1 & \beta_2 \\ 1 & 0 \end{pmatrix}}_{B} \begin{pmatrix} x_{t-1} \\ x_{t-2} \end{pmatrix} + \begin{pmatrix} \epsilon_t \\ 0 \end{pmatrix} \tag{3}$$

that is the matrix formulation of the difference equation in eq. (1)[1]. The eigenvalues are

$$\lambda_{\pm} = \frac{\beta_1 \pm \sqrt{\beta_1^2 + 4\beta_2}}{2} \,. \tag{4}$$

From standard dynamical systems theory, we know that if the eigenvalues $\lambda_\pm$ are complex conjugate the system shows spiral orbits whose stability is given by the conditions $|\lambda_\pm| < 1$. From (4) follows that these condition are satisfied if

$$\begin{cases} |\beta_1| < 2 \\ -1 < \beta_2 < -\frac{\beta_1^2}{4} \leq 0\,. \end{cases} \tag{5}$$

On the other hand, when $|\lambda_\pm| > 1$, the spiral orbits are unstable and tend towards infinity and in the particular case of $|\lambda_\pm| = 1$, the orbits lie on a circle rotating counterclockwise. The frequency of such oscillations is given by

$$\omega = \frac{1}{2\pi} \arccos\Big(\frac{\beta_1}{2\sqrt{-\beta_2}}\Big)\,.$$

2 Linear E-I circuit

Let E_t and I_t , the state variables of an excitatory and inhibitory population, respectively. The dynamics of a coupled E-I system can be described in the simplest form by a 2-dimensional linear difference equation of the first order

$$\begin{pmatrix} E_t \\ I_t \end{pmatrix} = W \begin{pmatrix} E_{t-1} \\ I_{t-1} \end{pmatrix} + \begin{pmatrix} \eta_e \\ \eta_i \end{pmatrix}\,, \tag{6}$$

where we let W be a matrix of coupling and recurrent weights

$$W \equiv \begin{pmatrix} w_{ee} & w_{ei} \\ w_{ie} & w_{ii} \end{pmatrix}\,, \tag{7}$$

and η_i and η_e are the external inputs to the respective population. Again we can describe the dynamics by studying the eigenstructure of W

$$\lambda^2 - \mathrm{Tr}(W)\lambda + \det(W) = 0 \tag{8}$$

and we obtain

$$\begin{aligned} \lambda_\pm &= \frac{(\mathrm{Tr}(W))}{2} \pm \frac{\sqrt{(\mathrm{Tr}(W))^2 - 4\det(W)}}{2} \\ &= \frac{(w_{ee} + w_{ii})}{2} \pm \frac{\sqrt{(w_{ee} - w_{ii})^2 + 4w_{ei}w_{ie}}}{2}\,. \end{aligned} \tag{9}$$

The system exhibits spiral trajectories if the eigenvalues are complex conjugates, thus

$$\begin{aligned} (w_{ii} - w_{ee})^2 + 4w_{ei}w_{ie} < 0 \implies \\ w_{ei}w_{ie} < -\frac{(w_{ii} - w_{ee})^2}{4}\,, \end{aligned} \tag{10}$$

which implies that the coupling weights between excitatory and inhibitory population have opposite signs

$$w_{ei}w_{ie} < 0\,. \tag{11}$$

For damped oscillatory behavior, it is further required that

$$\begin{cases} |\,\mathrm{Tr}(W)| < 2\,; \\ 1 > \det(W) > \frac{\mathrm{Tr}^2(W)}{4} \geq 0\,. \end{cases} \tag{12}$$

We can rewrite the latter expressions as

$$\begin{cases} -2 < w_{ee} + w_{ii} < 2\,; \\ w_{ee}w_{ii} - 1 > w_{ei}w_{ie} > -\frac{(w_{ii}-w_{ee})^2}{4} \geq 0\,, \end{cases} \tag{13}$$

which shows the constraints on the recurrent and coupling weights of the linear circuit. The frequency of oscillation is given by

$$\omega = \frac{1}{2\pi} \arctan\left(\frac{(w_{ee} - w_{ii})^2 + 4w_{ei}w_{ie}}{2(w_{ee} - w_{ii})}\right)\,.$$

3 AR(2) model to E-I circuit

Here we show that any second-order linear system of the form (1) can be written as in (6) by an affine transformation A , which maps (x_t, x_{t-1}) onto an excitatory E_t and an inhibitory population I_t. In particular, we discuss the case of complex conjugate eigenvalues in (4).

A general linear transformation A is given by

$$\begin{pmatrix} E_t \\ I_t \end{pmatrix} = \underbrace{\begin{pmatrix} a_1 & a_2 \\ a_3 & a_4 \end{pmatrix}}_{A} \begin{pmatrix} x_t \\ x_{t-1} \end{pmatrix}, \tag{14}$$

which is an affine transformation of the (x_t, x_{t-1}) space if A is non-singular. Note that we are transforming the space (x_t, x_{t-1}) into a 2-dimensional representation given by the E and I populations at the same timestep t. Given that x_t obeys the second-order equation (1) of the AR(2) model, we can rewrite (14) as

$$\begin{aligned} \begin{pmatrix} E_t \\ I_t \end{pmatrix} &= A \begin{pmatrix} x_t \\ x_{t-1} \end{pmatrix} = A\left(\begin{pmatrix} \beta_1 & \beta_2 \\ 1 & 0 \end{pmatrix} \begin{pmatrix} x_{t-1} \\ x_{t-2} \end{pmatrix} + \epsilon_t \begin{pmatrix} 1 \\ 0 \end{pmatrix} \right) = \\ &= ABA^{-1} \begin{pmatrix} E_{t-1} \\ I_{t-1} \end{pmatrix} + \epsilon_t \begin{pmatrix} a_1 \\ a_3 \end{pmatrix} = \\ &= \underbrace{V \begin{pmatrix} E_{t-1} \\ I_{t-1} \end{pmatrix}}_{intrinsic} + \underbrace{\epsilon_t \begin{pmatrix} a_1 \\ a_3 \end{pmatrix}}_{noisy\ drive} . \end{aligned} \tag{15}$$

This is the general form of a linear E-I dynamical system that we seek, where the matrix V defines the dynamical proprieties of this discrete time system. The stability of the system in (14) is given by the roots of the characteristic polynomial

$$\lambda^2 - \mathrm{Tr}(V)\lambda + \det(V) .$$

Given that V and B are similar matrices through the affine transformation $V = ABA^{-1}$ the two systems have the same characteristic polynomial and relative eigenvalues, that is $\mathrm{Tr}(V) = \beta_1$ and $\det(V) = -\beta_2$. Therefore, the eigenstructure of the system is the same as in (4)

$$\lambda_{\pm} = \frac{\beta_1}{2} \pm \frac{\sqrt{\beta_1^2 + 4\beta_2}}{2}, \tag{16}$$

and the dynamical proprieties of the system remain unchanged. In particular, if (1) exhibits stable oscillatory behavior, then so will the system in (14).

3.1 General form of the affine weights

We derive now the general form of the weights by explicitly computing V in (15)

$$\begin{aligned} V &= ABA^{-1} \\ &= \frac{1}{\det(A)} \begin{pmatrix} a_1 & a_2 \\ a_3 & a_4 \end{pmatrix} \begin{pmatrix} \beta_1 & \beta_2 \\ 1 & 0 \end{pmatrix} \begin{pmatrix} a_4 & -a_2 \\ -a_3 & a_1 \end{pmatrix} \\ &= \frac{1}{\det(A)} \begin{pmatrix} a_1\beta_1 + a_2 & a_1\beta_2 \\ a_3\beta_1 + a_4 & a_3\beta_2 \end{pmatrix} \begin{pmatrix} a_4 & -a_2 \\ -a_3 & a_1 \end{pmatrix} \\ &= \frac{1}{\det(A)} \begin{pmatrix} a_4(a_1\beta_1 + a_2) - a_1a_3\beta_2 & -a_2(a_1\beta_1 + a_2) + a_1^2\beta_2 \\ a_4(a_3\beta_1 + a_4) - a_3^2\beta_2 & -a_2(a_3\beta_1 + a_4) + a_1a_3\beta_2 \end{pmatrix} . \end{aligned} \tag{17}$$

Then (15) takes the form of

$$\begin{pmatrix} E_t \\ I_t \end{pmatrix} = \begin{pmatrix} v_{ee} & v_{ei} \\ v_{ie} & v_{ii} \end{pmatrix} \begin{pmatrix} E_{t-1} \\ I_{t-1} \end{pmatrix} + \epsilon_t \begin{pmatrix} a_1 \\ a_3 \end{pmatrix} \tag{18}$$

where

$$
\begin{aligned}
v_{ee} &= \frac{1}{\det(A)}\big[a_4(a_1\beta_1 + a_2) - a_1 a_3\beta_2\big] \\
v_{ei} &= \frac{1}{\det(A)}\big[-a_2(a_1\beta_1 + a_2) + a_1^2\beta_2\big] \\
v_{ie} &= \frac{1}{\det(A)}\big[a_4(a_3\beta_1 + a_4) - a_3^2\beta_2\big] \\
v_{ii} &= \frac{1}{\det(A)}\big[-a_2(a_3\beta_1 + a_4) + a_1 a_3\beta_2\big]
\end{aligned}
\tag{19}
$$

are the recurrence and coupling weights under the affine transformation (14).

3.2 Opposite sign of the coupling weights

Let (3) be the matrix form of the AR(2) model such that $\lambda_{\pm}$ are complex conjugates, then it can be shown that $v_{ie}v_{ei} < 0$ for all affine transformations A, that is two populations provide, respectively, negative and positive feedback to each other. Indeed, from (5) we can write the following inequalities for the numerators of the coupling weights in (19) as

$$
\begin{aligned}
v_{ei}: &\quad -a_2(a_1\beta_1 + a_2) + a_1^2\beta_2 < -\left(a_2^2 + a_1^2\frac{\beta_1^2}{4} + a_2 a_1\beta_1\right) \le 0 \\
v_{ie}: &\quad a_4(a_3\beta_1 + a_4) - a_3^2\beta_2 > a_4^2 + a_3^2\frac{\beta_1^2}{4} + a_4 a_3\beta_1 \ge 0\,,
\end{aligned}
\tag{20}
$$

which hold true $\forall\, a_{ij} = [A]_{ij}$. Both expressions have the same structure, and we note that they are strictly different from zero $\forall\, A$ such that

$$
a_i \neq -\frac{\beta_1}{2}a_j \tag{21}
$$

where $i = 1, 3$ and $j = 2, 4$, respectively. Therefore, for given β_1 and β_2, there always exist a specific choice of the affine transformation A giving one or both null coupling weights. Supplementary Note 1 Supplementary Figure 12 shows the distribution of the weights in the parameter space (β_1, β_2) for a given transformation A.

As expected, the spaces are divided into two regions of positive and negative weights by a straight line which is the solution of (19) for, respectively, every weight. A different choice of the affine transformation A corresponds to different orientations and positions of those lines in the (β_1, β_2) space.

The sign of the determinant $\det(A)$ switches the sign of both coupling weights thus preserving their relation, indeed $\forall\ A$ we have

$$
\begin{cases}
v_{ei} \ge 0\ ;\ v_{ie} \le 0 & \text{if } \det(A) > 0 \\[2ex]
v_{ei} \le 0\ ;\ v_{ie} \ge 0 & \text{if } \det(A) \le 0
\end{cases}
\tag{22}
$$

and thus

$$
v_{ei}v_{ie} = \frac{(-a_2(a_1\beta_2 + a_2) + a_1^2\beta_1)(a_4(a_3\beta_2 + a_4) - a_3^2\beta_1)}{\det^2(A)} < 0 \quad \forall\, A\,. \tag{23}
$$

The invariance to the sign of $\det(A)$ is strictly connected with the symmetry of interpreting the state variables of the transformation (14) as the excitatory or inhibitory population, that is having a respective positive or negative interaction weight on the other population.

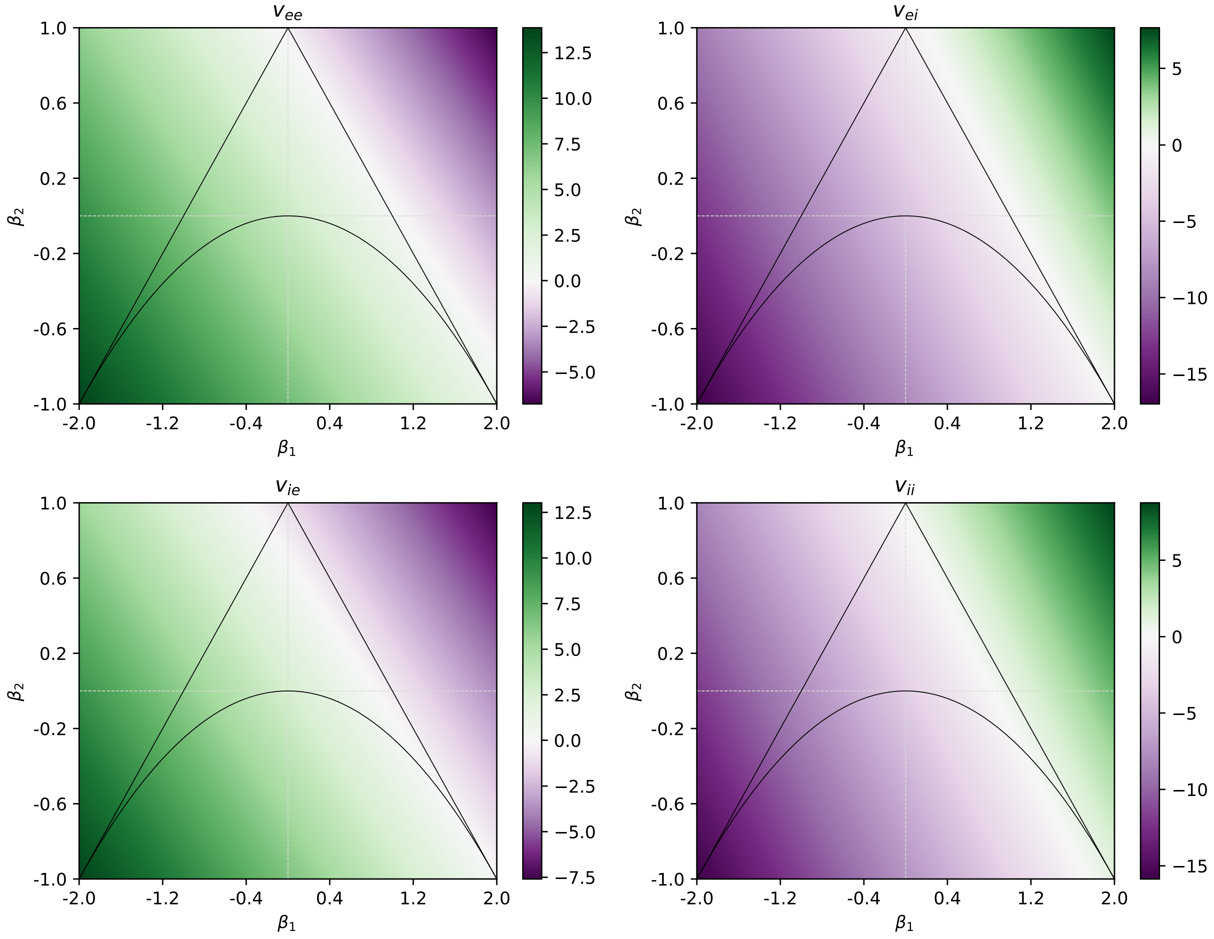

Supplementary Figure 12: **Distribution of the weights**. Values of v_{ee}, v_{ei}, v_{ie}, $v_{ii} \in V$ from equation (19) in the parameter space (β_1, β_2), for the affine transformation A: $a_1 = 1$, $a_2 = -1.1$, $a_3 = 1$, $a_4 = -0.84$. The parameter space is divided into regions based on the dynamical proprieties of the system in eq. (1)[1].

3.3 Affine transformation of the phase space

Note that the linear E-I circuit has the same canonical Jordan form of the AR(2), as the composition of two affine transformations is again an affine transformation[2]. Indeed, if J is the canonical form for the AR(2) system (3) through the invertible matrix P and the variable (w_t, w_{t-1}) such that

$$\begin{pmatrix} x_t \\ x_{t-1} \end{pmatrix} = P \begin{pmatrix} w_t \\ w_{t-1} \end{pmatrix} = PJ \begin{pmatrix} w_{t-1} \\ w_{t-2} \end{pmatrix} = PJP^{-1} \begin{pmatrix} x_{t-1} \\ x_{t-2} \end{pmatrix}$$

then we obtain

$$\begin{pmatrix} E_t \\ I_t \end{pmatrix} = A \begin{pmatrix} x_t \\ x_{t-1} \end{pmatrix} = AP \begin{pmatrix} w_t \\ w_{t-1} \end{pmatrix} = AP\, J\, (AP)^{-1} \begin{pmatrix} E_{t-1} \\ I_{t-1} \end{pmatrix}$$

thus the Jordan canonical form is the same.

We stress that the key step in the transformation (14) is mapping the (x_t, x_{t-1}) space into the affine space (E_t, I_t) with the same time-label t. In this way, both E_t and I_t are a linear combination of (x_t, x_{t-1}) which, together with the matrix B, is a sufficient description of the dynamics of an AR(2) as shown in (3). Supplementary Note 1 Supplementary Figure 13 shows the effect of warping the phase space with a specific choice of the affine transformation, and how the latter defines the dynamics of the (E_t, I_t) in the new phase space.

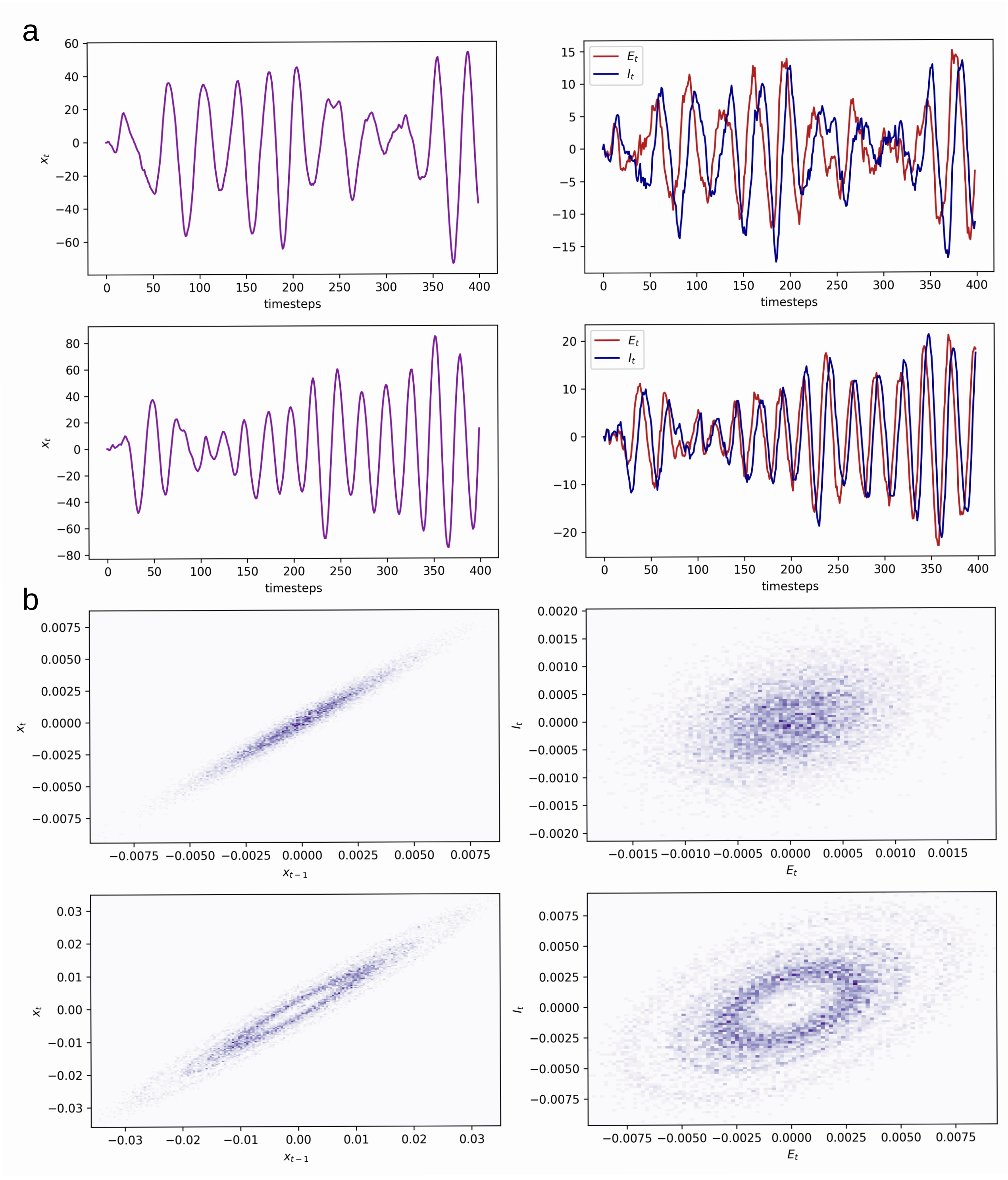

Supplementary Figure 13: **Transformation of the phase space**. a) Plots on the upper part show the dynamics of the time series x_t simulated from (1) and the relative affine transformation in the E_t and I_t traces for A: $a_1 = 1$, $a_2 = -1.1$, $a_3 = 1$, $a_4 = -0.84$ and $\beta_1 = 1.9449$ and $\beta_2 = -0.9801$. Plots in the bottom part shows the same dynamics with $\beta_1 = 1.9449$ and $\beta_2 = -1$. b) Plots on the upper part show the density in the (x_t, x_{t-1}) phase space and in the (E_t, I_t) phase space for $\beta_1 = 1.9449$ and $\beta_2 = -0.9801$, while the bottom plots show the same phase spaces for $\beta_1 = 1.9449$ and $\beta_2 = -1$, i.e. where the eigenvalue magnitude is equal to 1. We average 10000 numerical solutions with different noise realisations to obtain the phase space density. For eigenvalue magnitudes below 1, the phase plane shows a stable focus perturbed by stochastic noise fluctuations and damped oscillations. For an eigenvalue magnitude of 1 (critical point) the system exhibits stable oscillations perturbed by stochastic noise.

3.4 Choice of the affine transformation

Given the generality of our previous results, we now address how to constraints the possible choice of the affine transformation matrix A to account for specific requirements.

First, note that in (15), A transforms also the way the external noise term influences both the excitatory and inhibitory population. We want both variables to be influenced by the stochastic input in the same manner, and thus $a_1 = a_3$. This is based on the assumption that, in a neuronal circuit, both E and I cells will be driven by an external input. For example, in the visual system, both fast-spiking interneuron populations and excitatory neuron populations will be driven by bottom-up inputs triggered by visual stimulation. Any choice of a_1 would just scale equally the effect of noise on both variables, so we require for simplicity that $a_1 = a_3 = 1$. This is our first constraint on the possible values of the coefficients of A.

Second, we analyse how different choices of the remaining free parameters a_2 and a_4 affects the dynamics of the E-I circuit, as we want to constrain the possible values of the parameters to a regime which accounts for published literature on the E and I populations in area V1. In particular, here we show how different choices of a_4 and a_2 result in different weight matrices, different balance between the E and I populations and different phase-delays.

Supplementary Note 1 Supplementary Figure 14 and Supplementary Figure 9 show how the relative phase shift and the E-I balance of the system change in the (a_2,a_4) parameter space and outline the region of space in which the coupling weights have magnitude smaller than 1, which we consider as the physiologically realistic regime.

We can see that there are two main axes in the (a_2, a_4) parameter space, which determine the E-I balance and the relative phase between E and I. In particular, the specific choice of a_1 and a_3 shapes the parameter space as it defines a focal point $(a_2, a_4) = -(a_1, a_3)$ (purple dots in Supplementary Note 1 Supplementary Figure 14) from which the two main axes are determined. Different choices of a_1 and a_3 give different positions of the focal point and scale the regions of interest in the parameter space but do not change the structure of the latter. In particular, the E-I balance constrains the choice of a_2 and a_4 along the main axis passing through $(a_2, a_4) = -(a_1, a_3)$, and physiological phase delays require a_2 and a_4 to be close to the focal point. Indeed, the literature on E-I interactions in general reports that the phase between E and I is about 2-3ms[3–4], and we have measured this also for V1[5]. Examining the point of a 2.3 ms delay between E and I, we obtain $a_2 = -1.1$ and $a_4 = -0.84$, and $v_{ei} = -0.2$, $v_{ie} = 0.2$, $v_{ii} - 1 = -0.1$, $v_{ee} - 1 = 0.05$. (Note that the relevant weights for updating the gradients $E_{t-1} - E_t$ and $I_t - I_{t-1}$ are $v_{ee} - 1$ and $v_{ii} - 1$). This corresponds to a transformation of a signal with opposite signs for E and I, with approximately equal contributions of the E and the I variable, which is compatible with physiology. In this regime, we have recurrent inhibition in the I population and a slightly negative weight on the E population. The resulting E_t and I_t variables show balance at a slight delay (Supplementary Note 1 Supplementary Figure 13).

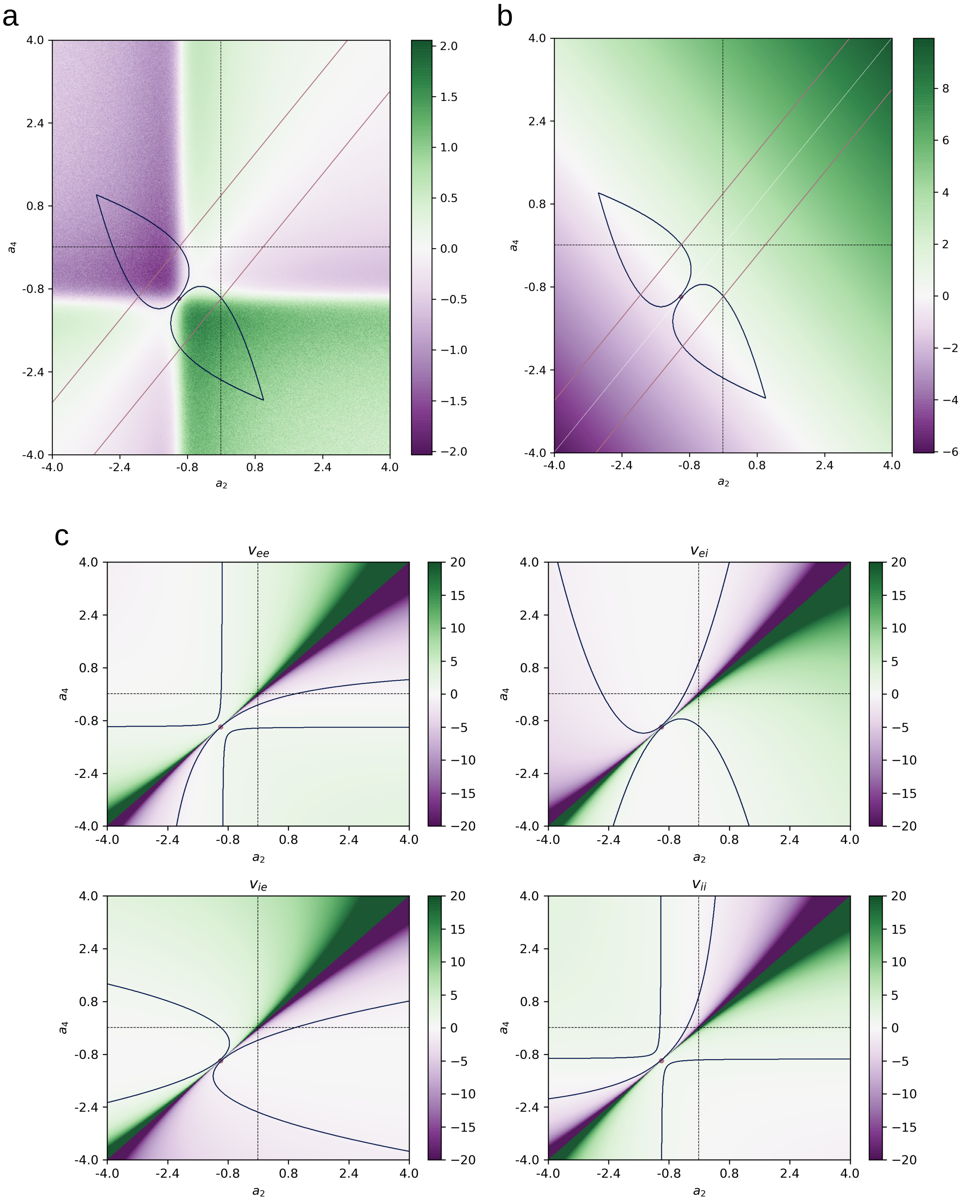

Supplementary Figure 14: **Phase shift and E-I balance in the (a_2, a_4) parameter space.** a) Phase shift (in radians) between I_t and E_t in the (a_2, a_4) space for fixed $a_1 = a_3 = 1$. We outline in blue the regions corresponding to magnitude of coupling weights smaller than one, while we outline in red the set of (a_2, a_4) corresponding to $\det(A) = \pm 1$. Note the symmetry defined by the line $a_2 = a_4$ corresponding to the symmetrical choice of the excitatory and inhibitory population as already discussed in section 3.2. Here the phase shift is computed as the difference of the mean angle between E_t and x_t and between I_t and x_t. b) E-I balance, which we define as the difference $v_{ie} - v_{ei}$, in the same parameter space as in panel A. The line passing by the point $(a_2, a_4) = -(a_3, a_1)$ defines the axes of E-I balance. Here the E-I balance is computed as the difference between the coupling weights v_{ei} and v_{ie}. c) How the weights change in the (a_2, a_4) parameter space. We outline in blue the region in which the respective weight has magnitude smaller than one. We set $\beta_1 = 1.9449$ and $\beta_2 = -0.9801$ for all the parameter space here shown.

SUPPLEMENTARY DISCUSSION

Stochastic cycle-by-cycle fluctuations in the frequency of gamma oscillations may have substantial ramifications for current theories involving gamma in inter-areal communication. Some degree of cycle-by-cycle variability would be required in order to resolve competition between two presynaptic populations, which converge on the same postsynaptic target population, through selective gamma coherence. This is the case because the presynaptic populations would show full coherence with each other if their respective gamma-oscillations were sinusoidal (i.e. they would be distinguishable from each other only if they differed in phase). Note that gamma-frequency variability in the timescale of a few cycles can be shared between a presynaptic and postsynaptic population, as it has been shown in macaque areas V1 and V2[6]. This frequency tracking can, in principle, mediate communication between neuronal populations in the absence of competition. However, stochasticity in gamma-cycle duration can, itself, introduce problems for the ability of a receiving population to distinguish between *multiple* senders. This can be illustrated in the following case: Bosman et al. (2012) (see also Grothe et al., 2012) found that selective attention increases gamma-coherence between the “attended” V1 site and V4, as compared to the “non-attended” V1 site and V4 (from about 0.05 to 0.09)[7, 8]. Bosman et al. hypothesized that this results from the reported 3-4Hz increase in the peak frequency of the gamma oscillation in the “attended” V1 site, which, in turn, leads to transient phase precession and entrainment of V4 by this faster oscillation. However, if the standard deviation of gamma-cycle frequencies is ~10Hz, as our findings demonstrate, it is unclear how the receiving population can distinguish between two competing sender-oscillations with a small difference in gamma-peak frequency, in a reasonably short time- window. This problem was partly raised by Akam & Kullmann (2012), who modelled selective information transmission in a feedforward system between multiple senders and one receiver[9]. They created a “best-case-scenario” for selective information transmission by modulating the receiver’s gain in a phase aligned manner (coherence of 1) with the rhythm in the selected sender. When oscillation frequencies showed substantial variability, relatively long integration windows were required to achieve selective information transmission. This held true both when selection was based on coherence differences (1 for selected sender vs. 0 for distractors), and when it was based on relatively small frequency differences between senders[9]. Random fluctuations in cycle-amplitude in noise-driven oscillations likely exacerbate this problem: This is because damped harmonic oscillators driven by noise can go through relatively prolonged periods where cycle amplitudes are relatively weak and the system is weakly oscillatory, which might result in a failure to selectively entrain a receiver. In spite of these issues, a recent computational model based on E/I circuits suggests that in the presence of substantial cycle-by-cycle variability, selective information transfer can occur during coherent gamma-bursts if the “attended” sender leads the “non-attended” sender *in phase*[10]. The results of different computational models likely depend on certain assumptions on the nature of gamma oscillations and the mechanisms underlying inter-areal gamma-synchronization, which do not necessarily hold true. For example it has been shown that oscillatory, inter-areal coherence between LFPs (or between spikes and LFPs) can arise as a byproduct of inter-areal connectivity and oscillations

in a sender, in the absence of any phase-coupling between oscillators or resonance[11-14]. Alternatively, V1-V4 gamma-synchronization might result from “entrainment” of the V4 population by V1 and might involve non-linear coupling or resonance mechanisms, which amplify inputs in certain frequencies. Once entrainment is established, it might subsequently make communication more effective[15]. However, it is unclear whether such a scenario applies to linear, damped harmonic oscillators driven by noise: If inputs from a sender are added as an input to a receiver that acts as a linear, damped harmonic oscillator, the interaction between the “state” of the receiver and the inputs from the sender will be linear, in contrast to what is supposed by non-linear mechanisms of entrainment.

Clarification by Pascal Fries

The previous paragraph is not endorsed by Pascal Fries, and he clarifies the following: The paragraph considers consequences of gamma-frequency variability for scenarios, in which selective neuronal communication is mediated by selective entrainment, as proposed in the Communication-through-Coherence (CTC) hypothesis [15]. The CTC hypothesis is supported by empirical evidence: When two stimuli induce two V1 gamma rhythms, and one stimulus is attended, primarily the attended V1 gamma entrains V4 gamma [7, 8], and primarily the attended stimulus determines V4 firing rates [16, 17]. Computational studies show that the selective entrainment is sufficient to explain the selective stimulus routing [10, 18, 19]. The previous paragraph discusses a hypothesis about how the selective entrainment might come about, namely that it might be related to an observed 3-4 Hz frequency increase of the attended V1 gamma [7, 15]. The paragraph argues that it would be unclear how a receiving population could distinguish between two sender oscillations with a 3-4 Hz frequency difference, given an overall gamma-frequency SD of 10 Hz, argueing that a computational study by Akam et al. [9] modeled similar conditions and found them to be insufficient. Here are some clarifications: (1) The crucial observation of Bosman et al. [7] is the selective V1-V4 entrainment, irrespective of whether it is related to the attentional increase in V1 gamma frequency or not. (2) The selective V4 firing-rate representation of the attended input [16, 17], with the selective V4 entrainment by the attended V1 gamma [7, 8], are empirical observations. They were obtained in the same awake-macaque model system as the data of the present paper, such that gamma-frequency variability is evidently not preventing them. In fact, Palmigiano et al. demonstrate that selective routing can be particularly effective with transient, variable-frequency bouts of oscillations and corresponding interareal entrainment [10]. (3) The previous paragraph does quickly mention the computational model by Palmigiano et al., but only to immediately proceed with argueing that some computational models likely depend on assumptions which do not necessarily hold true, and it refers to a recent preprint. This preprint argues that a) a rhythm in a sender is copied into the synaptic inputs at a receiver, and thereby explains sender-receiver coherence, b) receiver neurons need not entrain their spike output to this rhythm, and generally do not do this. However, this does not hold for the case that is dealt with in the present paper and the previous paragraph, namely the sender being V1 and the receiver being V4: a) V4 spikes are synchronized to the V4 gamma rhythm [4, 20-22], b) the V4 gamma rhythm is entrained by the V1 gamma rhythm [7, 8, 23], such that c) V4 spikes are entrained

by the V1 gamma rhythm [8]. Also, other studies recording simultaneously from several visual areas have shown clear interareal gamma-band spike synchronization [24-27]. (4) The observations of Bosman et al. cannot be explained by the mechanism proposed in this preprint, nor by the linear interactions between a receiver state and sender inputs discussed at the end of the previous paragraph. Bosman et al. found that two stimuli induce two V1 gamma rhythms, whose strengths are not modulated by attention. For this case, neither the simple copying mechanism nor the linear interaction can explain why Bosman et al. found that the attended V1 gamma, as compared to the equally strong unattended V1 gamma, shows much stronger coherence with V4. By contrast, computational models implementing selective neuronal entrainment can explain this [10, 19]. (5) The previous paragraph claims that Bosman et al. hypothesized that the selective entrainment of V4 by the attended V1 gamma results from the 3-4 Hz increase in the attended as compared to the unattended V1 gamma, yet it fails to mention that Bosman et al., and Fries et al [15], propose that this gamma-frequency increase combines with empirically observed theta-rythmic gamma-phase resetting [28, 29]. The cited models of Akam et al. did not consider theta-rhythmic gamma-phase resetting and therefore do not apply to the proposal of Bosman et al. and Fries et al.. (6) The previous paragraph juxtaposes empirically observed coherence values to simulated coherence values. However, empirically observed coherence values are most likely strongly underestimated because of measurement and estimation noise. Thus any direct comparison can be misleading. (7) Some sources of gamma-frequency variance likely do not hinder and maybe even support stimulus selection. One such source of gamma-frequency variance is the time after the last microsaccade [29]. This variance applies to all simultaneously presented stimuli and thereby does not interfere with the selection of one of them. Another such source of gamma-frequency variance is the time after stimulus onset (see Fig. 1 of the present paper). If a more recent onset of one stimulus lends the induced gamma a higher frequency, this might well implement the higher salience of recently appeared stimuli [15]. Thus, this variability is not a problem for frequency-based stimulus selection, but a potential mechanism thereof. In the Bosman et al. study, the two stimuli appeared simultaneously, equalizing onset-related salience, and precluding corresponding frequency differences.

Supplementary References

1. Hamilton, JD. Time Series Analysis. Princeton University Press (1994).

2. Arnold, VI. Geometrical methods in the theory of ordinary differential equations. Vol. 250. Springer Science & Business Media (2012).

3. Hasenstaub A, Shu Y, Haider B, Kraushaar U, Duque A, McCormick DA. Inhibitory postsynaptic potentials carry synchronized frequency information in active cortical networks. *Neuron* **47**, 423-435 (2005).

4. Vinck M, Womelsdorf T, Buffalo EA, Desimone R, Fries P. Attentional modulation of cell-class-specific gamma-band synchronization in awake monkey area v4. *Neuron* **80**, 1077-1089 (2013).

5. Onorato I*, et al.* A Distinct Class of Bursting Neurons with Strong Gamma Synchronization and Stimulus Selectivity in Monkey V1. *Neuron* **105**, 180-197 e185 (2020).

6. Roberts MJ*, et al.* Robust gamma coherence between macaque V1 and V2 by dynamic frequency matching. *Neuron* **78**, 523-536 (2013).

7. Bosman CA*, et al.* Attentional stimulus selection through selective synchronization between monkey visual areas. *Neuron* **75**, 875-888 (2012).

8. Grothe I, Neitzel SD, Mandon S, Kreiter AK. Switching neuronal inputs by differential modulations of gamma-band phase-coherence. *J Neurosci* **32**, 16172-16180 (2012).

9. Akam TE, Kullmann DM. Efficient "communication through coherence" requires oscillations structured to minimize interference between signals. *PLoS Comput Biol* **8**, e1002760 (2012).

10. Palmigiano A, Geisel T, Wolf F, Battaglia D. Flexible information routing by transient synchrony. *Nat Neurosci* **20**, 1014-1022 (2017).

11. Schneider M, Dann B, Sheshadri S, Scherberger H, Vinck M. A general theory of coherence between brain areas. *BioRxiv*, (2020).

12. Pesaran B*, et al.* Investigating large-scale brain dynamics using field potential recordings: analysis and interpretation. *Nat Neurosci* **21**, 903-919 (2018).

13. Buzsaki G, Schomburg EW. What does gamma coherence tell us about inter-regional neural communication? *Nat Neurosci* **18**, 484-489 (2015).

14. Schomburg EW*, et al.* Theta phase segregation of input-specific gamma patterns in entorhinal-hippocampal networks. *Neuron* **84**, 470-485 (2014).

15. Fries P. Rhythms for Cognition: Communication through Coherence. *Neuron* **88**, 220-235 (2015).

16. Reynolds JH, Chelazzi L, Desimone R. Competitive mechanisms subserve attention in macaque areas V2 and V4. *The Journal of neuroscience : the official journal of the Society for Neuroscience* **19**, 1736-1753 (1999).

17. Moran J, Desimone R. Selective attention gates visual processing in the extrastriate cortex. *Science* **229**, 782-784 (1985).

18. Buehlmann A, Deco G. Optimal information transfer in the cortex through synchronization. *PLoS computational biology* **6**, (2010).

19. Börgers C, Kopell NJ. Gamma oscillations and stimulus selection. *Neural computation* **20**, 383-414 (2008).

20. Fries P, Reynolds JH, Rorie AE, Desimone R. Modulation of oscillatory neuronal synchronization by selective visual attention. *Science* **291**, 1560-1563 (2001).

21. Fries P, Womelsdorf T, Oostenveld R, Desimone R. The effects of visual stimulation and selective visual attention on rhythmic neuronal synchronization in macaque area V4. *J Neurosci* **28**, 4823-4835 (2008).

22. Bichot NP, Rossi AF, Desimone R. Parallel and serial neural mechanisms for visual search in macaque area V4. *Science* **308**, 529-534 (2005).

23. Bastos AM*, et al.* Visual areas exert feedforward and feedback influences through distinct frequency channels. *Neuron* **85**, 390-401 (2015).

24. Engel AK, König P, Kreiter AK, Singer W. Interhemispheric synchronization of oscillatory neuronal responses in cat visual cortex. *Science* **252**, 1177-1179 (1991).

25. Engel AK, Kreiter AK, Konig P, Singer W. Synchronization of oscillatory neuronal responses between striate and extrastriate visual cortical areas of the cat. *Proceedings of the National Academy of Sciences of the United States of America* **88**, 6048-6052 (1991).

26. Smith MA, Jia X, Zandvakili A, Kohn A. Laminar dependence of neuronal correlations in visual cortex. *J Neurophysiol* **109**, 940-947 (2013).

27. Zandvakili A, Kohn A. Coordinated Neuronal Activity Enhances Corticocortical Communication. *Neuron* **87**, 827-839 (2015).

28. Bosman CA, Womelsdorf T, Desimone R, Fries P. A microsaccadic rhythm modulates gamma-band synchronization and behavior. *J Neurosci* **29**, 9471-9480 (2009).

29. Lowet E, Roberts MJ, Bosman CA, Fries P, De Weerd P. Areas V1 and V2 show microsaccade-related 3-4-Hz covariation in gamma power and frequency. *Eur J Neurosci* **43**, 1286-1296 (2016).
