# Supplementary material for: Spontaneous variability in gamma dynamics described by a damped harmonic oscillator driven by noise
Source: Nat Commun. 2022 Apr 19;13:2019. doi: 10.1038/s41467-022-29674-x (PMC9018758; doi:10.1038/s41467-022-29674-x)
Supplement: Supplementary file 2 — Reporting Summary [file 41467_2022_29674_MOESM2_ESM.pdf]

nature portfolio

Corresponding author(s): Georgios Spyropoulos, Martin Vinck, Pascal Fries

Last updated by author(s): Feb 13, 2022

Reporting Summary

Nature Portfolio wishes to improve the reproducibility of the work that we publish. This form provides structure for consistency and transparency in reporting. For further information on Nature Portfolio policies, see our Editorial Policies and the Editorial Policy Checklist.

Statistics

For all statistical analyses, confirm that the following items are present in the figure legend, table legend, main text, or Methods section.

n/a	Confirmed	
☐	☒	The exact sample size (*n*) for each experimental group/condition, given as a discrete number and unit of measurement
☐	☒	A statement on whether measurements were taken from distinct samples or whether the same sample was measured repeatedly
☐	☒	The statistical test(s) used AND whether they are one- or two-sided<br>*Only common tests should be described solely by name; describe more complex techniques in the Methods section.*
☐	☒	A description of all covariates tested
☐	☒	A description of any assumptions or corrections, such as tests of normality and adjustment for multiple comparisons
☐	☒	A full description of the statistical parameters including central tendency (e.g. means) or other basic estimates (e.g. regression coefficient) AND variation (e.g. standard deviation) or associated estimates of uncertainty (e.g. confidence intervals)
☐	☒	For null hypothesis testing, the test statistic (e.g. F, t, r) with confidence intervals, effect sizes, degrees of freedom and P value noted<br>*Give P values as exact values whenever suitable.*
☒	☐	For Bayesian analysis, information on the choice of priors and Markov chain Monte Carlo settings
☒	☐	For hierarchical and complex designs, identification of the appropriate level for tests and full reporting of outcomes
☐	☒	Estimates of effect sizes (e.g. Cohen's *d*, Pearson's *r*), indicating how they were calculated

Our web collection on statistics for biologists contains articles on many of the points above.

Software and code

Policy information about availability of computer code

Data collection	Recordings were made with Tucker Davis Technologies (TDT) systems, the Plexon system, and the Neuralynx system. Once collected, data was organized in standard FieldTrip data formats and is available upon reasonable request.
Data analysis	Data was analyzed using the MATLAB FieldTrip toolbox.

For manuscripts utilizing custom algorithms or software that are central to the research but not yet described in published literature, software must be made available to editors and reviewers. We strongly encourage code deposition in a community repository (e.g. GitHub). See the Nature Portfolio guidelines for submitting code & software for further information.

Data

Policy information about availability of data

All manuscripts must include a data availability statement. This statement should provide the following information, where applicable:

- Accession codes, unique identifiers, or web links for publicly available datasets
- A description of any restrictions on data availability
- For clinical datasets or third party data, please ensure that the statement adheres to our policy

The electrophysiological data generated in this study are under active use by the reporting laboratory; all data presented in this manuscript are available upon reasonable request. Source Data is provided for all figures.

Field-specific reporting

Please select the one below that is the best fit for your research. If you are not sure, read the appropriate sections before making your selection.

☒ Life sciences ☐ Behavioural & social sciences ☐ Ecological, evolutionary & environmental sciences

For a reference copy of the document with all sections, see nature.com/documents/nr-reporting-summary-flat.pdf

Life sciences study design

All studies must disclose on these points even when the disclosure is negative.

Sample size	The study followed up closely on the previous study by Atallah & Scanziani (2009, Neuron), which used n=6 rats, and performed similar analyses. We used n=6 monkeys for our study. Typical sample sizes in non-human primate studies are n=2 or n=3 monkeys. Thus, we exceed the typical sample size for non-human primate studies, and perform statistics across animals which allows for an inference to the population level, which is commonly lacking in non-human primate studies. We therefore obtain higher generalizability than is common in non-human primate studies.
Data exclusions	No animals or datasets were excluded from the analysis.
Replication	Initial analyses were performed in two monkeys. We then analyzed an additional four monkeys and replicated our findings for those initial two monkeys. We show that all animals have a significant correlation between gamma-cycle amplitude and duration, thus replicating our findings.
Randomization	We used within-subject permutation testing to generate a null hypothesis distribution. Thus, the same variability and correlation structure in the signals are preserved in our null distributions.
Blinding	Blinding was not a relevant aspect for the current study. There were no specific interactions of the experimenters with the animal subjects depending on the stimulus conditions, as the animals sit in a separate booth.

Reporting for specific materials, systems and methods

We require information from authors about some types of materials, experimental systems and methods used in many studies. Here, indicate whether each material, system or method listed is relevant to your study. If you are not sure if a list item applies to your research, read the appropriate section before selecting a response.

Materials & experimental systems

n/a	Involved in the study	
☒	☐	Antibodies
☒	☐	Eukaryotic cell lines
☒	☐	Palaeontology and archaeology
☐	☒	Animals and other organisms
☒	☐	Human research participants
☒	☐	Clinical data
☒	☐	Dual use research of concern

Methods

n/a	Involved in the study	
☒	☐	ChIP-seq
☒	☐	Flow cytometry
☒	☐	MRI-based neuroimaging

Animals and other organisms

Policy information about studies involving animals; ARRIVE guidelines recommended for reporting animal research

Laboratory animals	Adult macaque monkeys (macaca mulatta, 4 male, 2 female).
Wild animals	The study did not involve wild animals
Field-collected samples	The study did not involve samples collected from the field.
Ethics oversight	The experiments were approved by the responsible regional or local authority, which was the Regierungspräsidium Darmstadt, Germany, for monkeys H, I, J, L and T, and the ethics committee of the Radboud University, Nijmegen, Netherlands, for monkey P.

Note that full information on the approval of the study protocol must also be provided in the manuscript.
